# Association between Serum Uric Acid and Liver Enzymes in Adults Aged 20 Years and Older in the United States: NHANES 2005–2012

**DOI:** 10.3390/jcm12020648

**Published:** 2023-01-13

**Authors:** Hong Lin, Guoyou Dai, Song Huang, Zhaoyang Chen, Zhaohui Jin, Zhiyao He

**Affiliations:** 1 Department of Pharmacy, West China Hospital, Sichuan University, Chengdu 610041, China; 2 School of Clinical Medicine, Sichuan College of Traditional Chinese Medicine, Mianyang 621000, China; 3 Key Laboratory of Drug-Targeting and Drug Delivery System of the Education Ministry and Sichuan Province, Sichuan Research Center for Drug Precision Industrial Technology, West China School of Pharmacy, Sichuan University, Chengdu 610041, China

**Keywords:** serum uric acid, alanine aminotransferase, aspartate aminotransferase, liver enzyme, liver function, nonalcoholic fatty liver disease

## Abstract

Although the relationship between serum uric acid (SUA) and nonalcoholic fatty liver disease has been widely reported, the relationship between SUA and liver enzymes has rarely been reported. The purpose of this study was to evaluate the association of SUA levels with alanine aminotransferase (ALT) and aspartate aminotransferase (AST) in populations aged 20 years and older in the United States. We analyzed 7165 individuals aged 20 years and older from the National Health and Nutrition Examination Survey (NHANES) in the United States. Weighted multiple linear regression models were used to analyze the relationship between SUA and ALT and AST. A generalized additive model and a smooth curve fitting were used to observe the linear relationship. SUA was positively correlated with ALT and AST. In addition, the overall increasing trend of ALT and SUA was observed across the SUA quartile groups. In the stratified analysis by sex and race, the SUA levels in male, female, Mexican American, and Non-Hispanic White individuals, and those of another race, were positively correlated with ALT and AST. However, the SUA levels in Non-Hispanic Black individuals had a nonlinear relationship with ALT and AST. In individuals aged 20 years and older in the United States (excluding Non-Hispanic Black individuals), SUA levels were positively associated with ALT and AST. Therefore, with a rise in SUA levels, liver function should be monitored or intervened with in people aged 20 years and older in the United States.

## 1. Introduction

Serum uric acid (SUA) is the main end product of purine metabolism, usually in the form of urate. It is well known that approximately two-thirds of urate is excreted by the kidneys and the remainder is excreted extra-renally, while a dysfunction in extrarenal excretion might increase the risk of hyperuricemia (HUA) [1,2]. In recent years, the prevalence of HUA has shown a steady upward trend in different countries or regions around the world [3,4]. The prevalence of HUA has been stable in the United States from 2007 to 2016, and the prevalence of HUA was at 20.1% (approximately 47.1 million people) from 2015 to 2016 [5]. It has been reported that SUA is associated with a variety of diseases, such as cardiovascular disease [6,7], kidney disease [8], diabetes [9,10], etc. There might be a common genetic risk locus between SUA and these diseases [11].

The liver has complex physiological functions, including metabolism, secretion, bile excretion, detoxification, and so on [12,13]. Alanine aminotransferase (ALT) and aspartate aminotransferase (AST), commonly referred to as “liver enzymes”, mainly exist in hepatocytes and in catalyzed transamination for gluconeogenesis and amino acids [14]. When the liver is damaged, the cell membrane structure is disrupted and liver enzymes are released into the bloodstream, resulting in elevated serum enzyme levels [15]. Therefore, they are used to test liver function and are also specific markers for evaluating hepatic cell injury [16]. Elevated SUA levels might lead to liver dysfunction via various mechanisms for increasing liver fat accumulation [17,18,19]. Currently, nonalcoholic fatty liver disease (NAFLD) is identified as the major cause of liver disease and the most common cause of abnormal liver function, with an overall prevalence of NAFLD of approximately 25% worldwide [20]. It has been reported that NAFLD is closely associated with cardiovascular disease [21], chronic kidney disease [22], type 2 diabetes mellitus [23], and so on.

In recent years, a large number of studies have shown that the prevalence of NAFLD is closely related to levels of SUA [24]. A cross-sectional study of 8925 participants in China showed that the NAFLD risk increased with increasing SUA levels [25], and another prospective study also supported the above finding [26]. Recently, Wei et al. identified SUA levels as an independent predictive risk factor for NAFLD in a 4-year prospective study in China [27]. In addition, a cross-sectional study of 10,732 nondiabetic adults in the United States showed that the risk of developing NAFLD increased with increasing SUA levels, and this positive relationship was also observed even if SUA was at a normal level [28].

We believe that there might be a potential connection between SUA and liver enzymes. However, the relationship between SUA and liver enzymes has rarely been reported, and the underlying mechanism was not clear. A recent study in a Bangladeshi population found that SUA levels were positively correlated with ALT, while a significant correlation was not observed between SUA levels and AST [29]. Therefore, based on the data in the NHANES database, the present study was conducted to investigate the relationship between SUA levels and ALT and AST in people aged 20 years and older in the United States.

## 2. Materials and Methods

### 2.1. Data Sources

We used data from the National Health and Nutrition Examination Survey (NHANES) (https://www.cdc.gov/nchs/nhanes/, accessed on 29 July 2022), which contains representative health and nutrition data on the noninstitutionalized U.S. civilian population. The study used demographic data, dietary data, examination data, laboratory data, and questionnaire data. All studies in NHANES were approved by the Institutional Review Board of the National Center for Health Statistics (https://www.cdc.gov/nchs/nhanes/irba98.htm, accessed on 29 July 2022).

### 2.2. Study Population

**Figure 1:**
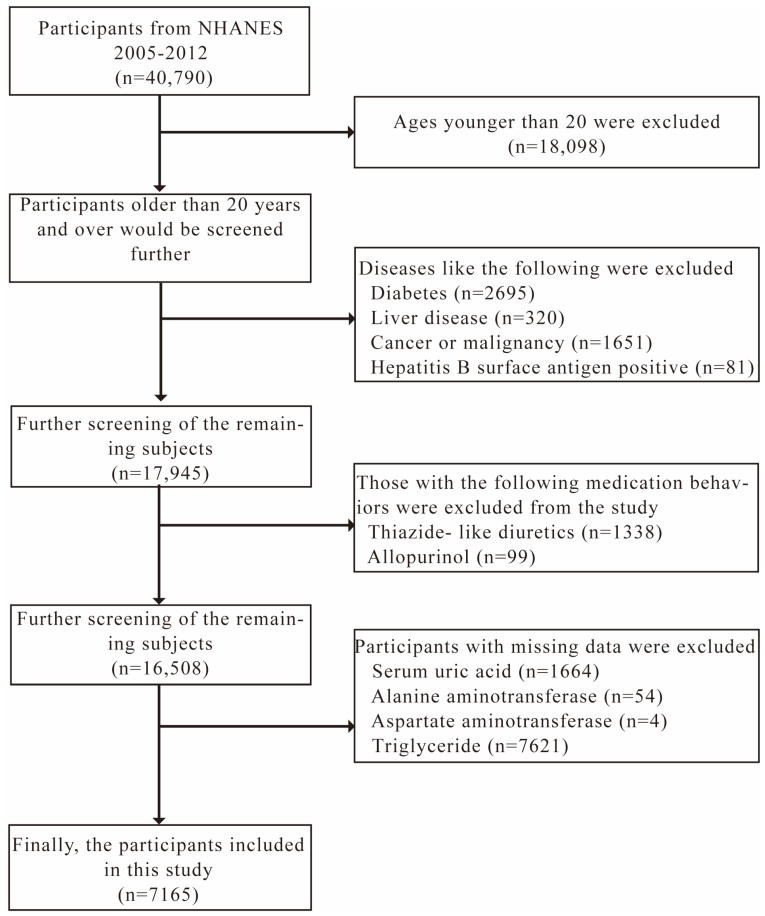
Flowchart of the participant screening process.

In this cross-sectional study, 4 periods of the NHANES (2005–2012) were selected, and 40,790 participants were enrolled (05–06: 10,348; 07–08: 10,149; 09–10: 10,537; 11–12: 9756). After excluding participants who were younger than 20 years (n = 18,098), who reported that they had diabetes (n = 2695), liver disease (n = 320), or cancer or malignancy (n = 1651), who had a positive blood test for the hepatitis B surface antigen (n = 81), who had used thiazide-like diuretics (n = 1338) or allopurinol (n = 99) within 1 month before the survey, and who lacked SUA (n = 1664), ALT (n = 54), AST (n = 4), or triglycerides (TG, n = 7621), a total of 7165 eligible subjects were enrolled as our analytic sample (Figure 1).

### 2.3. Study Variables

For this study, the exposure variable was SUA (mg/dL), and the outcome variables were ALT (U/L) and AST (U/L). SUA, ALT, and AST were measured with a Beckman Synchron LX20 between 2005 and 2007 and a Beckman UniCel DxC800 from 2008 to 2012. Hyperuricemia was defined as an SUA level >7 mg/dL in men and >6 mg/dL in women [30]. Serum ALT levels >40 U/L in males and >31 U/L in females and serum AST levels >37 U/L in males and >31 U/L in females were the cut-off values for the liver enzymes [31]. In addition, there were many covariates in this study, including age (years), sex (male or female), race (Mexican American, Non-Hispanic White, Non-Hispanic Black, or another race), education (less than high school, high school, or greater than high school), income to poverty ratio (less than 1.3, 1.3–3.5, or greater than 3.5), smoking behavior (never smoked, former smoker, or current smoker), drinking behavior (never drank, former drinker, moderate drinker, or heavy drinker), systolic blood pressure (SBP, mm Hg), diastolic blood pressure (DBP, mm Hg), body mass index (BMI, kg/m^2^), and waist circumference (WC, cm), and levels of serum total bilirubin (TBIL, mg/dL), serum creatinine (SCR, mg/dL), total cholesterol (TC, mg/dL), TG (mg/dL), high-density lipoprotein cholesterol (HDL-C, mg/dL), low-density lipoprotein cholesterol (LDL-C, mg/dL), glycated hemoglobin (HBA1C, %), platelets (PLT, 1000 cells/μL), and fasting blood glucose (FBG, mg/dL). It is worth noting that blood lipid levels were only investigated for participants who fasted for more than 8.5 h and less than 24 h. Details of all the variables are publicly available at https://www.cdc.gov/nchs/nhanes/ (accessed on 29 July 2022).

### 2.4. Statistical Analyses

We used R version (version 3.6.3, R Foundation for Statistical Computing^®^, Vienna, Austria) and EmpowerStats (X&Y Solutions, Inc., Boston, MA, USA, http://www.empowerstats.com, accessed on 1 August 2022) for the statistical analyses in this study. Continuous variables were displayed as a mean ± SD or median (IQR), and categorical variables were expressed as weighted percentages (%). A linear regression model was used for comparisons between continuous data, and the chi-square test was used for comparisons between categorical variables. First, the correlation of SUA with ALT and AST was analyzed in three different models using a weighted multiple linear regression model. Model 1 was an unadjusted variable model. Model 2 was adjusted for the recognized confounding factors (e.g., age, sex, and race). Model 3 was a model that was adjusted for all covariates, which were previously reported. SUA quartiles were grouped for analysis, and the median of the SUA quartiles was used as a continuous variable for the trend analysis, and SUA was grouped by hyperuricemia and normality. A stratified analysis by sex and race was also performed. Furthermore, to better display the relationship between SUA and ALT and AST, a generalized additive model and a smooth curve fitting plot were used to observe the linear or nonlinear relationship. Values were considered statistically significant if *p*-values were less than 0.05.

## 3. Results

### 3.1. Study Population Characteristics

In the cross-sectional population, 7165 subjects were enrolled. SUA was categorized into four quartiles (Q1: <4.4 mg/dL, Q2: 4.4–5.2 mg/dL, Q3: 5.3–6.1 mg/dL, and Q4: >6.1 mg/dL dL). The baseline characteristics of the subjects based on the SUA quartile are presented in Table 1 and Table 2. The results showed significant differences in all except the income-to-poverty ratio (*p* > 0.05, Table 1 and Table 2). It was found that the highest quartile grouping of SUA might be male, Non-Hispanic White, and heavy drinkers (Table 1). In addition, we observed that SUA increased with increases in SBP, DBP, BMI, WC, SCR, TC, TG, LDL-C, FBG, ALT, AST, the incidence of ALT elevation, and the incidence of AST elevation from Q1 to Q4 (*p* < 0.05), while HDD-C and PLT gradually decreased from Q1 to Q4 (*p* < 0.05) (Table 2). In addition, the overall prevalence of HUA was 15.7%, the incidence of ALT elevation was 12.2%, and the incidence of AST elevation was 8.5%.

### 3.2. Association of SUA with ALT and AST

**Figure 2:**
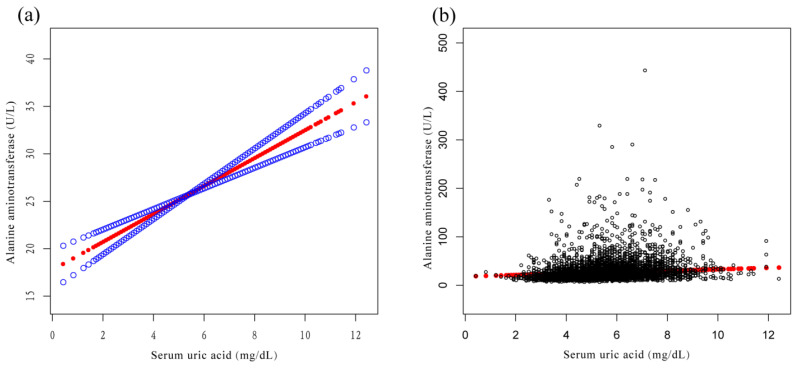
Relationship between serum uric acid and liver enzymes. (**a**) Relationship between serum uric acid and alanine aminotransferase (R^2^ = 0.150). The red line represents the fitted curve, and the blue line represents the 95% confidence interval. (**b**) Each black point represents a sample, and the red line represents the fitted curve.

**Figure 3:**
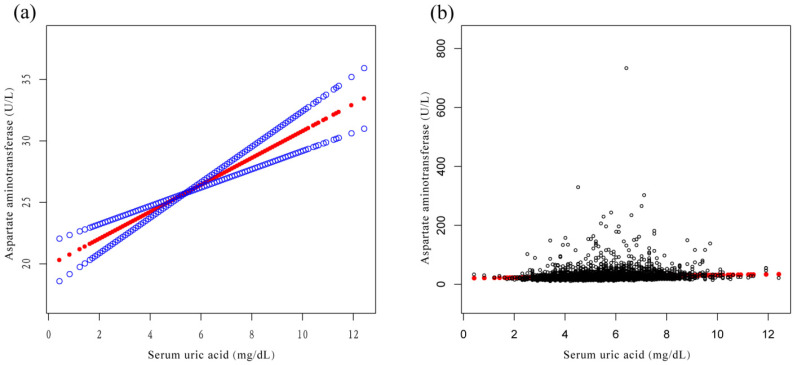
Relationship between serum uric acid and liver enzymes. (**a**) Relationship between serum uric acid and aspartate aminotransferase (R^2^ = 0.077). The red line represents the fitted curve, and the blue line represents the 95% confidence interval. (**b**) Each black point represents a sample, and the red line represents the fitted curve.

**Figure 4:**
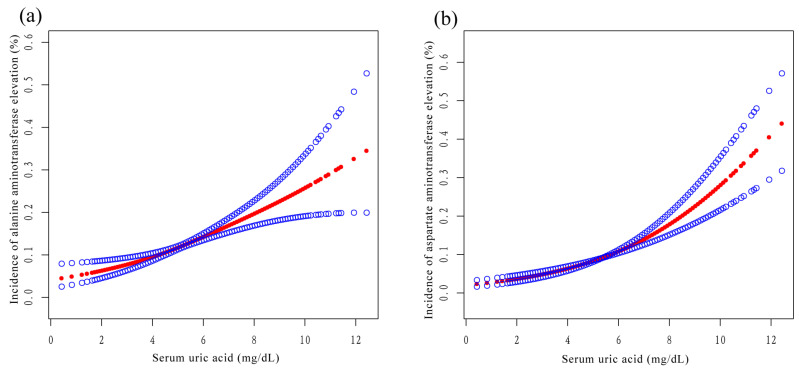
Relationship between serum uric acid and rate of liver enzyme elevations. (**a**) Relationship between serum uric acid and incidence of alanine aminotransferase elevation. (**b**) Relationship between serum uric acid and incidence of aspartate aminotransferase elevation. The red line represents the fitted curve, and the blue line represents the 95% confidence interval.

A weighted multiple linear regression model was used to evaluate the relationship between SUA and ALT and AST in the three models. In the three models, SUA was positively correlated with ALT (Table 3) and AST (Table 4). In model 3: SUA was positively correlated with ALT (β = 1.5, 95% CI: 1.1, 1.8) and positively correlated with AST (β = 1.1, 95% CI: 0.7, 1.4). Compared with Q1, ALT and AST of Q4 increased by 4.0 U/L and 2.9 U/L, respectively. The overall increasing trends of ALT and AST were significant in the three models (*p* < 0.001). In addition, smooth curve fitting diagrams of the relationship between SUA and ALT and AST are shown (Figure 2 and Figure 3). Figure 4a,b demonstrates the relationship of SUA with the incidence of ALT elevation and the incidence of AST elevation. We also performed a Pearson’s correlation analysis on SUA and liver enzymes, and SUA was still positively correlated with ALT and AST (Supplementary Figures S1 and S2).

### 3.3. Stratified Analysis according to Sex

**Figure 5:**
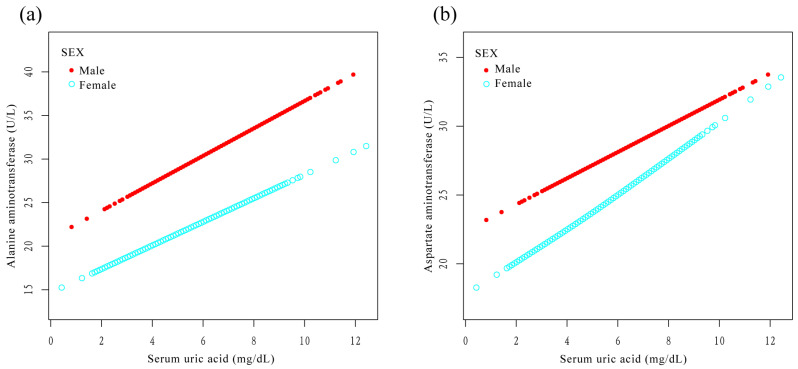
Relationship between serum uric acid and liver enzymes stratified by sex. The lines represent fitted curves. (**a**) Relationship between serum uric acid and alanine aminotransferase stratified by sex (R^2^ = 0.149). (**b**) Relationship between serum uric acid and aspartate aminotransferase stratified by sex (R^2^ = 0.077).

After stratification by sex, SUA was positively associated with ALT and AST in the three models. In model 3, SUA was positively associated with ALT in males (β = 1.2, 95% CI: 0.6, 1.8) and in females (β = 1.4, 95% CI: 1.0, 1.8) (Table 3). SUA was also positively correlated with AST in males (β = 0.9, 95% CI: 0.4, 1.4) and in females (β = 1.1, 95% CI: 0.7, 1.4) (Table 4). After stratification by sex, SUA plotted a smooth curve fit plot with ALT (Figure 5a) and AST (Figure 5b).

### 3.4. Stratified Analysis according to Race

**Figure 6:**
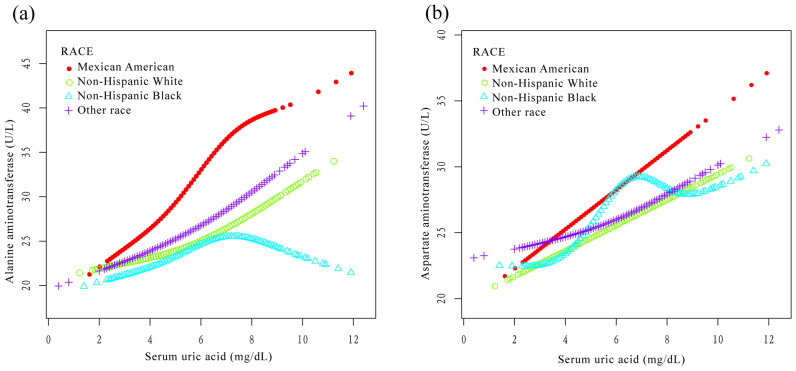
Relationship between serum uric acid and liver enzymes stratified by race. The lines represent fitted curves. (**a**) Relationship between serum uric acid and alanine aminotransferase stratified by race. (**b**) Relationship between serum uric acid and aspartate aminotransferase stratified by race.

After stratification by race, SUA was positively correlated with ALT in Mexican American individuals (β = 2.9, 95% CI: 1.5, 4.3), Non-Hispanic White individuals (β = 1.4, 95% CI: 0.9, 1.9), and those of other races (β = 1.5, 95% CI: 0.7, 2.4), except Non-Hispanic Black individuals (β = 0.7, 95% CI: −0.2, 1.5) (Table 3). In addition, SUA was also positively correlated with AST in Mexican American individuals (β = 2.0, 95% CI: 1.0, 2.9), Non-Hispanic White individuals (β = 1.0, 95% CI: 0.6, 1.4), and those of other race (β = 0.8, 95% CI: 0.2, 1.4), except Non-Hispanic Black individuals (β = 1.1, 95% CI: −0.1, 2.4) (Table 4). As shown in Figure 6a,b, after stratification by race, the SUA of Non-Hispanic Black individuals showed an obvious nonlinear relationship with ALT and AST, displaying an inverted U-shaped curve.

## 4. Discussion

In recent years, the association between SUA and NAFLD has been widely reported, while reports on SUA and liver enzymes are rare. Therefore, the purpose of our study was to investigate whether SUA was independently associated with ALT and AST in the U.S. population aged 20 years and older. There were two findings in this study. First, SUA was positively correlated with ALT and AST. SUA was also associated with the incidence of ALT elevation and AST elevation. The above associations were independent of all covariates. Second, the stratification analysis found that the SUA levels in Non-Hispanic Black individuals exhibited a nonlinear relationship with ALT and AST.

Previous studies have shown that SUA levels are associated with the prevalence of NAFLD. A 5-year retrospective cohort study in Korea indicated that the risk of NAFLD increased with increasing SUA levels [32]. In another cross-sectional study of 10,732 non-diabetic adults in the United States, elevated SUA levels were significantly associated with the risk of NAFLD diagnosed via ultrasound [28]. In addition, a cross-sectional study of 7569 participants in China showed that the risk of NAFLD increased significantly with increasing levels of SUA, and the probability was slightly higher in women than in men [33]. A cross-sectional study of 82,608 participants aged 20–60 in Israel manifested that SUA was positively associated with the incidence of elevated ALT, and women were at a greater risk of elevated ALT than men [34]. These results were consistent with the results of our study. However, a cross-sectional study of Bangladeshi adults reported that there was a positive association between SUA and ALT, but not SUA and AST [29]. Similarly, a prospective study of 3822 participants in China also showed a positive association between the incidence of increased ALT and SUA levels, but such a positive association was not found between the incidence of increased AST and SUA levels [35]. This contradicts the results of the association between SUA and AST in our study. Therefore, these contradictions are worthy of discussion and further study. The reason might be that the differences in region or race or covariates produce inconsistencies.

Existing studies show that there might be multiple mechanisms by which SUA caused liver dysfunction. Choi et al. suggested that uric acid-induced triglyceride accumulation could be mediated via a sterol regulatory element-binding protein 1 cleavage and nuclear translocation via induction of endoplasmic reticulum stress in hepatocytes [17]. Lanaspa et al. demonstrated that the production of SUA was accompanied by the generation of mitochondrial oxidants [18]. Mitochondrial oxidative stress resulted in a decreasing activity of aconitase in the Krebs cycle and an increasing accumulation of citrate, which stimulated the generation of hepatic fatty, resulting in hepatic dysfunction [18]. Furthermore, some scholars showed that fructose-induced uric acid could regulate the activity of AMP-activated protein kinase and AMP deaminase 2, thereby disrupting fatty acid oxidation and stimulating hepatic lipogenesis [19]. Based on the results of this study and these mechanisms, SUA might be an important factor leading to liver dysfunction.

It was worth noting that we found that the relationship between SUA and ALT and AST showed a nonlinear relationship in Non-Hispanic Black individuals (an inverted U-shaped curve, as seen in Figure 5). There was a segmentation effect of SUA in Non-Hispanic Black individuals: when SUA reached a certain value, ALT and AST did not increase with an increasing SUA. Currently, to our knowledge, there are no interethnic studies of SUA and liver enzymes in the United States. It has been reported that Non-Hispanic Black individuals have a protective effect against NAFLD, which might be related to genetic factors. However, studies have shown that patatin-like phospholipase domain-containing 3 gene sequence variation could lead to liver damage in Non-Hispanic Black individuals [36,37]. Therefore, prospective studies of different ethnic groups in the United States are needed in the future to elucidate the association and mechanism of SUA with liver enzymes in Non-Hispanic Black individuals.

Our study used a nationally representative sample from the United States, and the results were similar to the overall population. Furthermore, this is the first study to report the association between SUA and ALT and AST in Non-Hispanic Black individuals. However, there were some limitations of our study that deserve discussion. First, this study was a cross-sectional design, and there might be differences and biases in different covariates, which did not clarify their potential causality. Second, patients with cancer, diabetes, drug use behavior, and so on were excluded from this study. Therefore, we were not sure whether our findings were applicable to these special populations. In conclusion, further studies on the underlying mechanisms and prospective studies with large samples are needed to determine the exact mechanism of the association between SUA and ALT and AST.

## 5. Conclusions

SUA was positively associated with ALT and AST in adults 20 years and older in the United States, independent of covariates. However, the SUA levels in Non-Hispanic Black individuals showed a non-linear relationship with both ALT and AST. When SUA reached a certain value, there was a negative correlation. Therefore, it is necessary to monitor or intervene in the liver function of people with elevated uric acid levels. However, this relationship may not apply to Non-Hispanic Black individuals. Therefore, it is necessary to improve screening, prevention, and intervention in patients with elevated uric acid levels in order to reduce the economic burden in the future.

## Figures and Tables

**Table 1:** Population characteristics data of study participants.

Variable Features	Total	Serum Uric Acid Quartile Grouping				*p*-Value
		Q1 (<4.4 mg/dL)	Q2 (4.4–5.2 mg/dL)	Q3 (5.3–6.1 mg/dL)	Q4 (>6.1 mg/dL)	
Age, mean ± SD (years)	43.4 ± 15.5	41.5 ± 15.1	43.0 ± 15.2	44.9 ± 15.5	44.3 ± 15.9	<0.001
Sex, n (%)						<0.001
Male	49.4	10.9	35.2	64.5	81.9	
Female	50.6	89.1	64.8	35.5	18.1	
Race, n (%)						<0.001
Mexican American	9.4	10.9	9.6	8.8	8.4	
Non-Hispanic White	68.0	63.7	68.3	68.9	70.7	
Non-Hispanic Black	10.3	10.6	11.2	9.7	9.7	
Other race	12.3	14.8	11.0	12.6	11.2	
Education, n (%)						0.002
<High school	17.7	16.3	18.1	18.6	17.6	
High school	22.6	21.4	20.0	24.5	24.2	
>High school	59.7	62.3	61.7	56.9	58.0	
Missing	0.1	0	0.2	0	0.2	
Income to poverty ratio, n (%)						0.085
<1.31	20.4	22.3	21.1	20.6	18.0	
1.31–3.50	34.0	33.7	33.9	33.9	34.6	
>3.50	40.0	38.3	40.3	40.1	41.2	
Missing	5.6	5.7	4.8	5.5	6.2	
Smoking behavior, n (%)						<0.001
Never smoked	54.9	60.5	57.1	52.6	50.2	
Former smoker	21.8	18.5	20.4	21.5	26.1	
Current smoker	23.2	20.9	22.3	25.8	23.7	
Missing	0.1	0.1	0.1	0	0	
Drinking behavior, n (%)						<0.001
Never drank	16.8	19.7	16.9	16.3	14.8	
Former drinker	10.2	13.1	11.1	8.9	8.0	
Moderate drinker	55.7	50.5	55.3	58.5	58.1	
Heavy drinker	9.0	6.2	7.8	9.4	12.2	
Missing	8.2	10.6	8.9	6.9	6.9	

Continuous variables were used as means ± SD or medians (IQR), and *p*-values were calculated using a weighted linear regression model. Percentages were used for categorical variables, and *p*-values were calculated using a weighted chi-square test.

**Table 2:** Healthcare diagnosis data for study participants.

Variable Features	Total	Serum Uric Acid Quartile Grouping				*p*-Value
		Q1 (<4.4 mg/dL)	Q2 (4.4–5.2 mg/dL)	Q3 (5.3–6.1 mg/dL)	Q4 (>6.1 mg/dL)	
SBP, mean ± SD, mm Hg	119.2 ± 15.7	114.4 ± 15.6	117.4 ± 14.8	120.3 ± 14.8	123.9 ± 15.9	<0.001
DBP, mean ± SD, mm Hg	69.8 ± 10.8	67.0 ± 9.8	68.8 ± 10.0	70.4 ± 11.0	72.6 ± 11.2	<0.001
BMI, mean ± SD, kg/m^2^	28.0 ± 6.3	25.5 ± 5.3	27.1 ± 5.6	28.7 ± 6.0	30.4 ± 6.8	<0.001
WC, mean ± SD, cm	96.4 ± 15.2	88.5 ± 13.3	93.2 ± 13.2	98.8 ± 14.0	103.9 ± 15.2	<0.001
TBIL, mean ± SD, mg/dL	0.8 ± 0.3	0.7 ± 0.2	0.8 ± 0.3	0.8 ± 0.3	0.9 ± 0.3	<0.001
SCR, mean ± SD, mg/dL	0.9 ± 0.3	0.7 ± 0.2	0.8 ± 0.3	0.9 ± 0.2	1.0 ± 0.2	<0.001
TC, mean ± SD, mg/dL	196.6 ± 40.3	192.5 ± 38.4	196.5 ± 42.4	197.5 ± 40.1	199.6 ± 40.0	<0.001
TG, median (IQR), mg/dL	105.0 (75.0–154.0)	85.0 (62.5–125.0)	98.0 (71.0–142.0)	112.0 (80.0–162.0)	126.0 (91.0–185.0)	<0.001
HDD-C, mean ± SD, mg/dL	54.5 ± 15.8	62.3 ± 16.3	56.6 ± 15.1	52.0 ± 14.3	48.1 ± 13.7	<0.001
LDL-C, mean ± SD, mg/dL	116.9 ± 34.2	110.2 ± 31.8	116.5 ± 35.5	119.4 ± 34.8	120.8 ± 33.5	<0.001
HBA1C, mean ± SD, %	5.4 ± 0.5	5.3 ± 0.6	5.4 ± 0.6	5.4 ± 0.5	5.4 ± 0.5	<0.001
PLT, mean ± SD, 1000 cells/μL	253.1 ± 64.9	259.3 ± 69.4	256.4 ± 67.4	251.2 ± 62.7	246.4 ± 59.6	<0.001
FBG, mean ± SD, mg/dL	98.98 ± 16.48	95.1 ± 18.3	98.0 ± 19.0	100.4 ± 13.3	101.9 ± 13.9	<0.001
ALT, median (IQR), U/L	21.0 (16.0–28.0)	17.0 (14.0–22.0)	19.0 (16.0–25.0)	22.0 (17.0–31.0)	25.0 (19.0–36.0)	<0.001
AST, median (IQR), U/L	23.0 (19.0–27.0)	21.0 (18.0–24.0)	22.0 (19.0–26.0)	24.0 (20.0–28.0)	25.0 (21.0–31.0)	<0.001
Incidence of ALT elevation, n (%)	12.2	6.5	8.6	13.5	19.2	<0.001
Incidence of AST elevation, n (%)	8.5	5.9	6.3	8.6	12.3	<0.001

SBP, systolic blood pressure; DBP, diastolic blood pressure; BMI, body mass index; WC, waist circumference; TBIL, serum total bilirubin; SCR, serum creatinine; TC, total cholesterol; TG, triglyceride; HDL-C, high-density lipoprotein cholesterol; LDL-C, low-density lipoprotein cholesterol; HBA1C, glycated hemoglobin; PLT, platelet; FBG, fasting blood glucose; ALT, alanine aminotransferase; AST, aspartate aminotransferase. Continuous variables were used as means ± SD or medians (IQR), and *p*-values were calculated using a weighted linear regression model. Percentages were used for categorical variables, and *p*-values were calculated using a weighted chi-square test.

**Table 3:** The relationship between serum uric acid (mg/dL) and alanine aminotransferase (U/L).

	Model 1		Model 2		Model 3	
	β (95% CI)	*p*-Value	β (95% CI)	*p*-Value	β (95% CI)	*p*-Value
SUA per 1 mg/dL increase	3.6 (3.3, 3.9)	<0.001	2.5 (2.2, 2.9)	<0.001	1.5 (1.1, 1.8)	<0.001
SUA IV group						
Q1	Reference		Reference		Reference	
Q2	3.0 (1.9, 4.2)	<0.001	1.7 (0.5, 2.8)	0.004	0.4 (−0.7, 1.6)	0.475
Q3	7.6 (6.5, 8.8)	<0.001	4.5 (3.3, 5.8)	<0.001	2.1 (0.8, 3.4)	0.001
Q4	12.0 (10.9, 13.2)	<0.001	7.8 (6.6, 9.1)	<0.001	4.0 (2.6, 5.4)	<0.001
*p* for trend	<0.001		<0.001		<0.001	
Sex						
Male	2.7 (2.1, 3.3)	<0.001	2.8 (2.2, 3.4)	<0.001	1.2 (0.6, 1.8)	<0.001
Female	1.9 (1.5, 2.2)	<0.001	2.0 (1.6, 2.3)	<0.001	1.4 (1.0, 1.8)	<0.001
Race						
Mexican American	6.0 (5.0, 7.1)	<0.001	4.3 (3.0, 5.6)	<0.001	2.9 (1.5, 4.3)	<0.001
Non-Hispanic White	3.5 (3.1, 3.9)	<0.001	2.5 (2.0, 2.9)	<0.001	1.4 (0.9, 1.9)	<0.001
Non-Hispanic Black	2.6 (1.9, 3.2)	<0.001	1.3 (0.5, 2.0)	0.001	0.7 (−0.2, 1.5)	0.133
Other race	3.8 (3.1, 4.5)	<0.001	2.9 (2.0, 3.7)	<0.001	1.5 (0.7, 2.4)	<0.001

**Table 4:** The relationship between serum uric acid (mg/dL) and aspartate aminotransferase (U/L).

	Model 1		Model 2		Model 3	
	β (95% CI)	*p*-Value	β (95% CI)	*p*-Value	β (95% CI)	*p*-Value
SUA per 1 mg/dL increase	1.9 (1.6, 2.1)	<0.001	1.3 (1.0, 1.6)	<0.001	1.1 (0.7, 1.4)	<0.001
SUA IV group						
Q1	Reference		Reference		Reference	
Q2	1.4 (0.5, 2.4)	0.004	0.6 (−0.4, 1.6)	0.211	0.5 (−0.5, 1.5)	0.303
Q3	3.6 (2.6, 4.5)	<0.001	1.7 (0.6, 2.8)	0.002	1.4 (0.3, 2.5)	0.011
Q4	6.0 (5.1, 7.0)	<0.001	3.6 (2.5, 4.7)	<0.001	2.9 (1.7, 4.1)	<0.001
*p* for trend	<0.001		<0.001		<0.001	
Sex						
Male	1.3 (0.8, 1.8)	<0.001	1.3 (0.8, 1.8)	<0.001	0.9 (0.4, 1.4)	0.001
Female	1.3 (1.0, 1.6)	<0.001	1.1 (0.8, 1.5)	<0.001	1.1 (0.7, 1.4)	<0.001
Race						
Mexican American	2.9 (2.2, 3.6)	<0.001	2.3 (1.4, 3.1)	<0.001	2.0 (1.0, 2.9)	<0.001
Non-Hispanic White	1.7 (1.4, 2.0)	<0.001	1.2 (0.8, 1.5)	<0.001	1.0 (0.6, 1.4)	<0.001
Non-Hispanic Black	2.4 (1.4, 3.4)	<0.001	1.4 (0.3, 2.5)	0.016	1.1 (−0.1, 2.4)	0.075
Other race	1.6 (1.1, 2.0)	<0.001	1.3 (0.7, 1.9)	<0.001	0.8 (0.2, 1.4)	0.007

## Data Availability

Data supporting reported results can be found in the National Health and Nutrition Examination Survey (NHANES) (https://www.cdc.gov/nchs/nhanes/, accessed on 29 July 2022).

## Supplementary Materials

The following supporting information can be downloaded at: https://www.mdpi.com/article/10.3390/jcm12020648/s1, Figure S1: Analysis of Pearson correlation between serum uric acid and alanine aminotransferase; Figure S2: Analysis of Pearson correlation between serum uric acid and aspartate aminotransferase.
