# Supplementary material for: Association between Serum Uric Acid and Liver Enzymes in Adults Aged 20 Years and Older in the United States: NHANES 2005–2012
Source: J Clin Med. 2023 Jan 13;12(2):648. doi: 10.3390/jcm12020648 (PMC9864736; doi:10.3390/jcm12020648)
Supplement: Supplementary file 1 [file jcm-12-00648-s001.zip › jcm-2067886-supplementary.pdf]

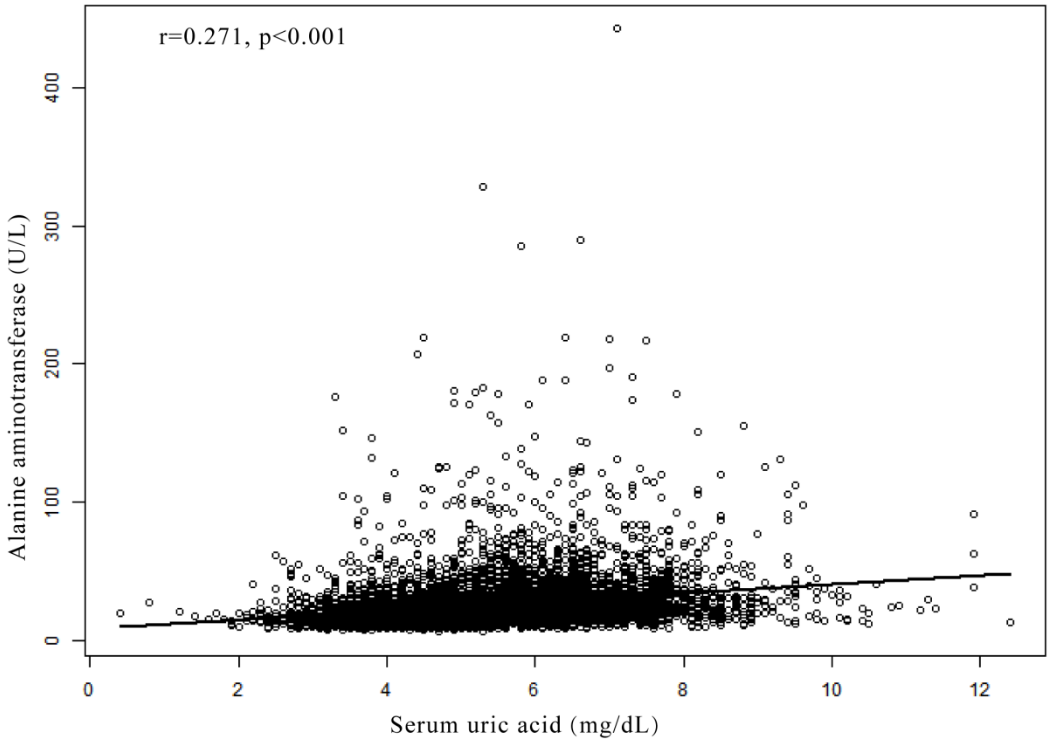

Figure S1. Analysis of Pearson correlation between serum uric acid and alanine aminotransferase.

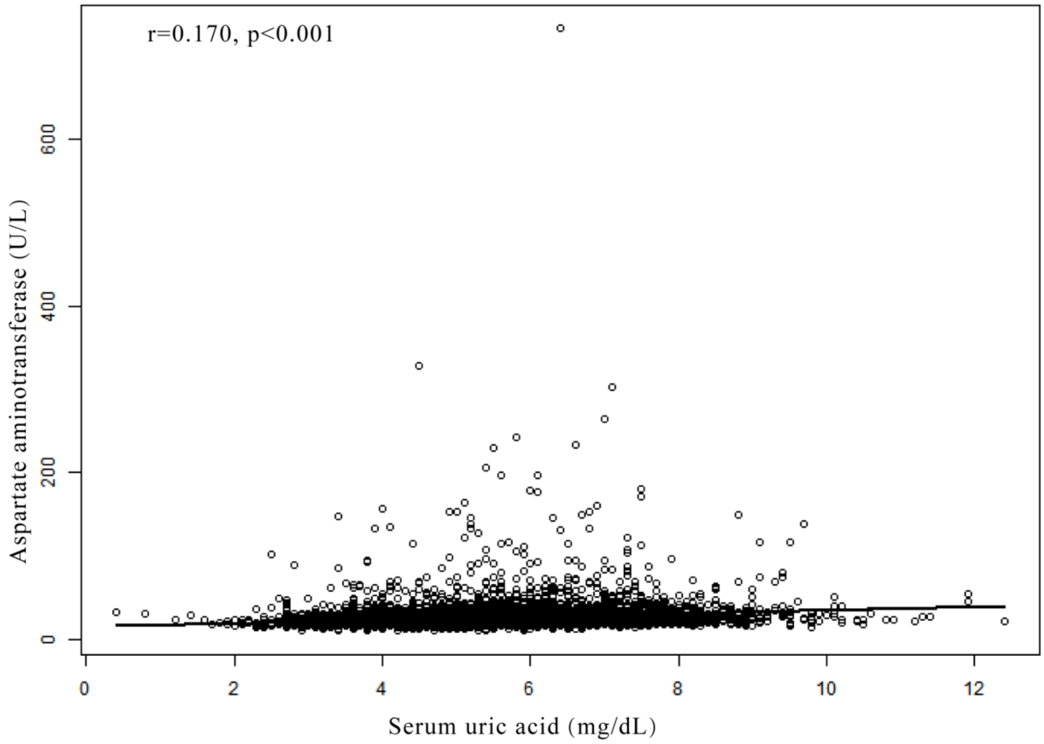

Figure S2. Analysis of Pearson correlation between serum uric acid and aspartate aminotransferase.
